# Post-prostatectomy rehospitalisation rates and risk factors in South Australian men with prostate cancer: evidence from linked data

**DOI:** 10.1007/s11255-025-04691-z

**Published:** 2025-07-25

**Authors:** Tenaw Tiruye, Alex Jay, Michael O’Callaghan, Liesel M. FitzGerald, David Roder, Kerri Beckmann

**Affiliations:** 1 Cancer Epidemiology and Population Health Research Group, Allied Health and Human Performance, University of South Australia, North Terrace, SAHMRI Building, Adelaide, SA 5000 Australia; 2 School of Public Health, Debre Markos University, Debre Markos, Amhara, Ethiopia; 3 Flinders Medical Centre, Bedford Park, SA Australia; 4 South Australian Prostate Cancer Clinical Outcomes Collaborative, Adelaide, SA Australia; 5 Flinders Health and Medical Research Institute, Flinders University, Adelaide, SA Australia; 6 Faculty of Health and Medical Sciences, University of Adelaide, Adelaide, SA Australia; 7 Menzies Institute for Medical Research, University of Tasmania, Hobart, TAS Australia

**Keywords:** Prostate cancer, Radical prostatectomy, Readmission, Rehospitalisation, Surgery

## Abstract

**Purpose:**

Prostate cancer is a common malignancy in men, with radical prostatectomy (RP) being a major treatment option. This study investigates post-prostatectomy rehospitalisation rates and risk factors in a cohort of South Australian men who underwent RP from 2002 to 2021 (n = 5105).

**Methods:**

Post-prostatectomy rehospitalisation rates at 30 and 90 days were measured from hospital discharge data, with reasons determined from ICD-10 codes. Rates per 1000 person-time were estimated, accounting for the length of follow-up. Zero inflated negative binomial regression analyses were used to identify sociodemographic and clinical factors associated with the number of hospital encounters following RP.

**Results:**

Approximately 13% of patients had at least one hospital visit within 90 days post-prostatectomy. Common reasons for early rehospitalisation (within 30 days) were urinary obstruction (3.2%), haematuria (2.6%), and urinary tract infection (2.5%). Older age (aged 75 + vs < 60: incidence rate ratio (IRR) 2.23, 95% CI: 1.88–2.64), highest comorbidity burden (3 + vs 0: IRR 2.33, 95% CI: 1.80–3.01), and high risk clinical characteristics (PSA > 20 vs < 10 ng/mL: IRR 1.67, 95% CI: 1.34–2.08 and Gleason score 9–10 vs < 7: IRR 1.39, 95% CI: 1.06–1.84) were associated with higher rehospitalisation rates. Conversely, men who were treated from 2016–2021 had 39% lower rehospitalisation rates (IRR 0.61, 95% CI: 0.53–0.71) compared with patients treated from 2002–2005.

**Conclusion:**

These findings highlight the importance of considering patient characteristics and tailoring post-surgical care plans to minimise rehospitalisation. The reduction in rehospitalisation over time may reflect advancements in surgical techniques, better patient selection or improved surgeon experience.

## Introduction

Prostate cancer is the most commonly diagnosed cancer in Australian men and for Australia overall, with an estimated 26,400 cases diagnosed in 2024 [1]. Radical prostatectomy (RP) is the most common treatment option for men with localised prostate cancer. Although prostate cancer survival rates are high, the 5-year survival rate exceeds 96% [1, 2], treatments can have a substantial impact on quality of life.

The impact of RP on patient-reported outcomes measures (PROMs), particularly sexual and urinary functions, is well documented [2–12]. The ProtecT trial has shown the greatest decline in sexual function and urinary continence in men who underwent RP [3]. Due to its surgical nature, RP could also cause other acute and chronic perioperative and postoperative complications that necessitate hospitalisation and additional medical interventions [13, 14]. Due to this, readmission rates have become a focal point of healthcare scrutiny due to their significant impact on overall healthcare expenditures [15]. Many studies that report 30- and 90-day postoperative complications are based on primary data collection (gathering data directly from a first-hand source), and complications are graded using the Clavien–Dindo classification system [16]. When primary data collection is not possible and/or not feasible, using administrative claims data provides an alternative means to measure the real-world experience of men treated with RP. Moreover, identifying the factors contributing to post-prostatectomy complications and hospitalisations can be instrumental in pinpointing patients at higher risk for these adverse events. In this study, we aimed to quantify the rates of rehospitalisation in men who underwent RP for prostate cancer and identify associated sociodemographic and clinical factors.

## Methods

### Study population

The study cohort included 5105 South Australian men who were treated with RP for prostate cancer between 2002 and 2021. These men were identified from the South Australian Cancer Registry (SACR) and the South Australian Prostate Cancer Clinical Outcomes Collaborative (SA-PCCOC) registry. Only men who underwent RP within a SA public hospital, identified through data linkage with the Integrated South Australian Activity Collection (ISAAC) were included. ISAAC contains data about patients separated (discharged) from hospitals [17]. Men who had radiotherapy within 90 days of the date of RP (before or after) were excluded.

### Measurement and variables

The outcome of interest (rehospitalisation) was defined as any complication, intervention, and/or medical procedure that required subsequent hospital care after discharge for RP, excluding perioperative events that co-occurred during the initial admission for RP. Perioperative events were analysed and reported separately. In consultation with a urologist, 24 toxicities/adverse effects potentially related to RP and that could lead to subsequent admission were identified. Reasons for admission were extracted from diagnosis codes using ISAAC ICD-10 codes, which are provided in Table 1. Outcomes were measured at 30 days and 90 days post-prostatectomy to accommodate early toxicities likely related to RP.

**Table 1:** Reasons for rehospitalisation from ICD-10 diagnosis codes within Integrated South Australian Activity Collection (ISAAC)

Reason/s	ICD-10 diagnosis codes
Cystitis	N30, N413
Urinary incontinence	R32, N393, N394, N328
Urinary tract infection	N390
Haematuria	R31, N02
Urinary obstruction	N320, N358, N359, N991, R33
Post-procedural genitourinary complications (not elsewhere classified)	N994, N995, N9989, N999
Urinary device complication	T830, T831, T834, T835, T836, T838, T839, Y846, Z436
Enteritis	K520, K521, K528, K529
Anorectal toxicities^‡^	K625, K628, K60, K61, K622, 623, K624, K626
Functional gastrointestinal changes^‡‡^	K590, K591, K594, K598, R191, R192, R194, R195, R14, R15, K599, R198
Anaemia	D500, D508, D509, D519, D529, D539, D59, D611, D619, D62, D648, D649
Thrombocytopenia	D691, D692, D693, D695, D696, D699
Neutropenia	D70
Infusion/transfusion	T80
Failed/difficult intubation	T8841, T8842
Haemorrhage/hematoma	T810
Puncture/laceration	T812
Foreign body	T815, T816
Medical-surgical complications (not elsewhere classified)	T811, T817, T818, T819, T88
Disruption of wound	T813
Wound infection	T814
Post-procedural circulatory complications	I97
Post-procedural respiratory complications	J95
Post-procedural gastrointestinal complications	K91

*ICD* International Classification of Diseases

^‡^Rectal bleeding, proctitis (excluding radiation proctitis K627), anorectal fistula, anorectal abscess, anorectal prolapse, anorectal stricture, anorectal ulcer

^‡‡^Constipation, diarrhoea, anal spasm, atony of colon, abnormal bowel sounds, hyperperistalsis, change in bowel habit, faecal abnormalities, flatulence, faecal incontinence and unspecific (K599, R198)

Potential predictor variables included age at diagnosis, socioeconomic status (SES), remoteness index, country of birth, year of treatment, Rx-Risk comorbidity index, diagnostic prostate-specific antigen (PSA) level and Gleason score. Age was categorised as < 60, 60–64, 65–69, 70–74 and ≥ 75 years. SES was derived from the Australian Bureau of Statistics (ABS) Socio-Economic Indices for Areas (SEIFA) scores, applied at the postal area level [18], and was categorised from lowest to highest quintile of socioeconomic advantage. Remoteness index was classified according to the Accessibility/Remoteness Index of Australia (ARIA) 2016 as major city, inner regional, outer regional, remote and very remote, indicating relative access to services according to postcode of residence [19]. Country of birth was extracted from the ABS country of birth standards [20] and grouped as ‘Australia and New Zealand’, ‘UK and Ireland’ and ‘Other’. Year of treatment (RP date) was grouped as 2002–2005, 2006–2010, 2011–2015 and 2016–2021. The Rx-Risk comorbidity index, which is based on prescription drug use data and has previously been validated in our cohort [21–23], was used to identify existing comorbidities in the year prior to prostate cancer diagnosis. Rx-Risk scores were grouped as 0, 1, 2 and ≥ 3 as previously recommended [21]. Diagnostic PSA levels (ng/mL) were grouped as < 10, 10–20 and > 20 ng/mL, whilst Gleason scores were grouped according to the 2014 International Society of Urological Pathology (ISUP) grading classification [24].

### Statistical analyses

The frequencies and percentages for each of the 24 selected reasons for rehospitalisation were calculated at 30 days and 90 days post-RP and differences across strata within variables were assessed using Chi-square tests. Rehospitalisation rates per 1000 person-time were calculated, with rates adjusted for variable lengths of follow-up and censoring due to death. We also reported the top ten reasons for rehospitalisation, the trend of rehospitalisation within 30 days following RP, and the most common perioperative events that occurred during the initial admission for RP.

Zero inflated negative binomial logistic regression models, which are used to model over-dispersed count variables with excessive zeros, were applied to assess the association between socioeconomic or clinical factors and rehospitalisation within 90 days post-prostatectomy. In the final models, adjusted for all potential covariates mentioned above, missing data for SES and remoteness index (< 1%) were imputed using random hot-deck imputation, whereas an extra category was generated to accommodate missing data for tumour characteristics (PSA and GG, 51% and 31% missing, respectively). All analyses were performed using Stata version 18 software (StataCorp).

### Ethics approval

Ethics approval was obtained from the South Australia Department for Health and Wellbeing Human Research Ethics Committee (HREC/20/SAH/58) and Australian Institute of Health and Welfare Ethics Committee (EO2020/5/1202). The requirement for informed consent was waived by the same ethics committees that approved the study (South Australia Department for Health and Wellbeing Human Research Ethics Committee and the Australian Institute of Health and Welfare Ethics Committee).

## Results

A total of 5105 men were included in the analyses: mean age at diagnosis of 68 (range 36 to 97) years. The majority resided in major cities (63%) and were born in Australia and New Zealand (65%). Of those with known tumour characteristics, the largest proportion had a PSA level of < 10 (32%) and Gleason score < 7 (33%). Patients who had rehospitalisation 90 days post-prostatectomy were older (mean age, 70 vs. 67 years), had higher comorbidity burden (Rx-Risk score 3 +, 50% vs. 40%) and born outside of Australia and New Zealand (39% vs. 35%) than patients who did not have rehospitalisation (all *p* < 0.001) (Table 2).

**Table 2:** Patient characteristics (N = 5105)

Variables		Total		Had readmission within 90 days of RP				*p* value
				No		Yes		
		No	%	No	%	No	%	
Total		5105	100	4425	86.7	680	13.3	
Age at diagnosis	< 60	1061	20.8	969	21.9	92	13.5	< 0.001
	60–64	887	17.4	795	18.0	92	13.5	
	65–69	1072	21.0	937	21.2	135	19.9	
	70–74	751	14.7	653	14.8	98	14.4	
	75 +	1334	26.1	1071	24.2	263	38.7	
	Mean ± standard deviation	67.8 ± 9.9		67.3 ± 9.8		71.4 ± 10.3		
Socioeconomic status	Lowest (most disadvantage)	1156	22.6	1000	22.6	156	22.9	0.001
	Low	1052	20.6	890	20.1	162	23.8	
	Average	765	15.0	645	14.6	120	17.6	
	High	1002	19.6	869	19.6	133	19.6	
	Highest (least disadvantage)	1112	21.8	1006	22.7	106	15.6	
	Missing^†^	18	0.4					
Remoteness index	Major city	3209	62.9	2776	62.7	433	63.7	0.590
	Inner regional	749	14.7	657	14.8	92	13.5	
	Outer regional	920	18.0	793	17.9	127	18.7	
	Remote/very remote	220	4.3	194	4.4	26	3.8	
	Missing^†^	7	0.1					
Place of birth	Australia and New Zealand	3295	64.5	2883	65.2	412	60.6	0.025
	UK and Ireland	842	16.5	707	16.0	135	19.9	
	Other	968	19.0	835	18.9	133	19.6	
Year of treatment	2002–2005	906	17.7	751	17.0	155	22.8	< 0.001
	2006–2010	1866	36.6	1676	37.9	190	27.9	
	2011–2015	1377	27.0	1191	26.9	186	27.4	
	2016–2021	956	18.7	807	18.2	149	21.9	
Rx-Risk comorbidity index	0	1468	28.7	1296	29.3	172	25.3	< 0.001
	1	861	16.9	771	17.4	90	13.2	
	2	684	13.4	604	13.6	80	11.8	
	3 +	2092	41.0	1754	39.6	338	49.7	
Diagnostic PSA level (ng/mL)	< 10	1641	32.1	1469	33.2	172	25.3	< 0.001
	10–20	512	10.0	449	10.1	63	9.3	
	> 20	329	6.4	259	5.9	70	10.3	
	Missing	2623	51.4	2248	50.8	375	55.1	
Gleason grade (GG) group	GG1 (score < 7)	1705	33.4	1488	33.6	217	31.9	< 0.001
	GG2 (score 3 + 4)	955	18.7	866	19.6	89	13.1	
	GG3 (score 4 + 3)	382	7.5	336	7.6	46	6.8	
	GG4 (score 8)	243	4.8	209	4.7	34	5.0	
	GG5 (score 9–10)	258	5.1	203	4.6	55	8.1	
	Missing	1562	30.6	1323	29.9	239	35.1	

*PSA* prostate-specific antigen, † not summarised due to data use agreement specifying not to report < 6 observations in a cell

**Fig. 1:**
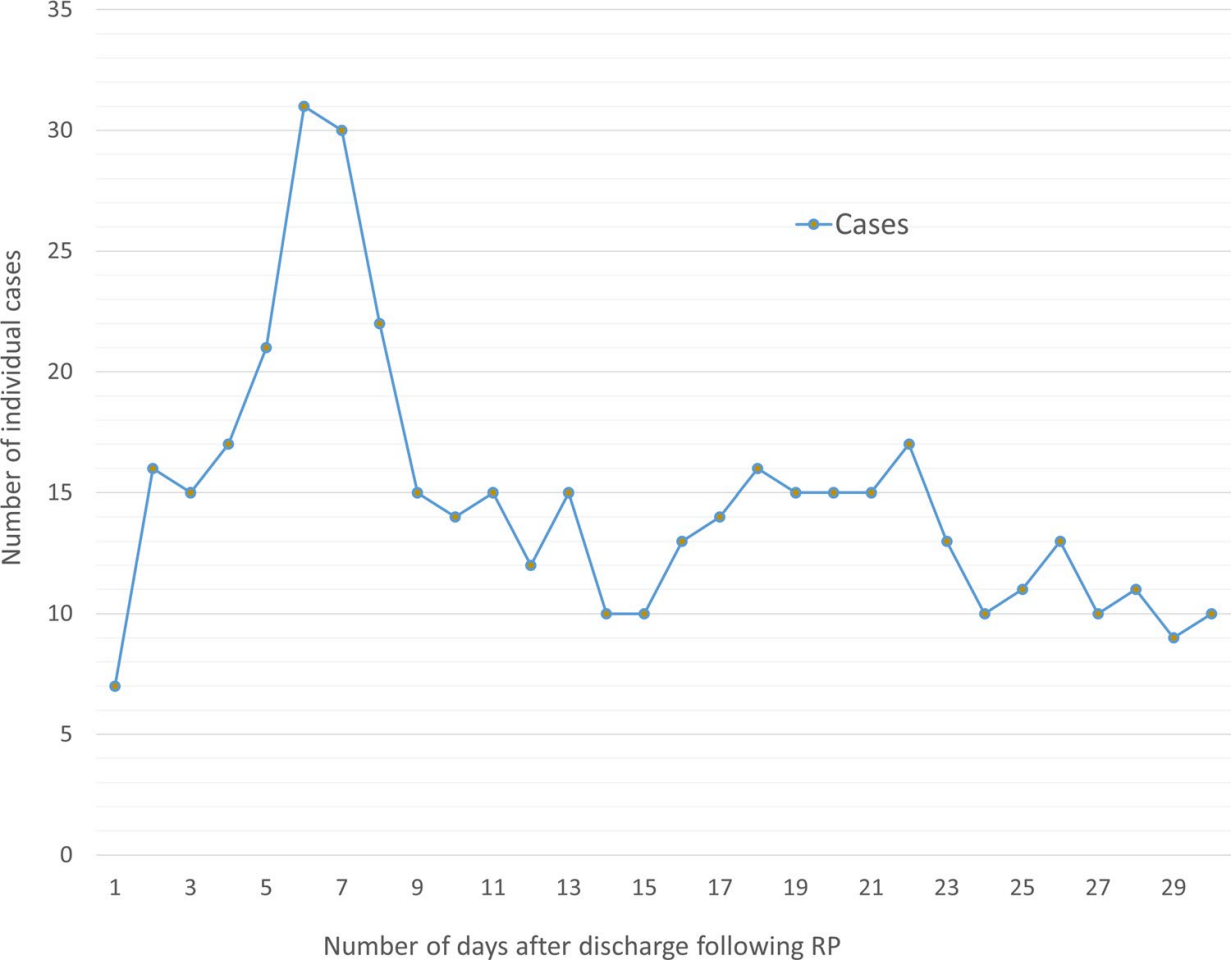
Trend of rehospitalisation within 30 days of RP. The trend was truncated at 30 days because of data use agreement not to report < 6 observations

Table 3 summarises rates for any rehospitalisation and for each reason of rehospitalisation. Overall, the cumulative rate of rehospitalisation 30 days post-prostatectomy was 87 per 1000 persons increasing to 135 per 1000 person by 90 days. The top ten reasons for rehospitalisation were urinary obstruction, haematuria, urinary tract infection, post-procedural genitourinary complications, urinary incontinence, urinary device complication, haemorrhage/hematoma, functional gastrointestinal changes and anaemia, with the number of events slightly different at 30 and 90 days (Tables 4 and 5). Rehospitalisation numbers increased during the first 6 to 7 days post-prostatectomy, then remained relatively consistent after day nine (Fig. 1). The most common perioperative events during the same admission included incontinence (4.7%), urinary tract infection (3.6%), haematuria (2.9%), post-procedural genitourinary complications (2.3%) and haemorrhage/haematoma (2.0%) (Table 6).

**Table 3:** Reasons for readmission post-prostatectomy and rate per 1000 person-time (N = 5105)

Reasons^†^	30 days				90 days			
	No	Rate^‡^	95% CI		No	Rate^§^	95% CI	
Cystitis	< 6	–	–	–	10	2.0	1.1	3.7
Urinary incontinence	59	11.6	9.0	15.0	93	18.4	15.0	22.6
Urinary tract infection	126	24.8	20.8	29.5	213	42.2	36.9	48.3
Haematuria	131	25.8	21.7	30.6	178	35.3	30.5	40.9
Urinary obstruction^#^	163	32.1	27.5	37.4	273	54.1	48.1	60.9
Post-procedural genitourinary complications (NEC)	103	20.3	16.7	24.6	167	33.1	28.5	38.5
Urinary device complications	56	11.0	8.5	14.3	82	16.3	13.1	20.2
Enteritis	< 6	–	–	–	10	2.0	1.1	3.7
Anorectal toxicities^††^	< 6	–	–	–	< 6	–	–	–
Functional gastrointestinal changes	47	9.2	6.9	12.3	77	15.3	12.2	19.1
Anaemia	46	9.1	6.8	12.1	94	18.6	15.2	22.8
Thrombocytopenia	< 6	–	–	–	6	1.2	0.5	2.7
Neutropenia	< 6	–	–	–	< 6	–	–	–
Infusion/transfusion	< 6	–	–	–	< 6	–	–	–
Failed/difficult intubation	< 6	–	–	–	< 6	–	–	–
Haemorrhage/haematoma	52	10.2	7.8	13.4	59	11.7	9.1	15.1
Puncture/laceration	< 6	–	–	–	< 6	–	–	–
Foreign body	< 6	–	–	–	< 6	–	–	–
Medical-surgical complications NEC	14	2.8	1.6	4.7	17	3.4	2.1	5.4
Disruption of wound	12	2.4	1.3	4.2	15	3.0	1.8	4.9
Wound infection	22	4.3	2.9	6.6	28	5.6	3.8	8.0
Post-procedural circulatory complications	16	3.2	1.9	5.1	24	4.8	3.2	7.1
Post-procedural respiratory complications	< 6	–	–	–	< 6	–	–	–
Post-procedural gastrointestinal complications	23	4.5	3.0	6.8	29	5.8	4.0	8.3
Any (at least one reason from the list above)	442	86.9	79.2	95.4	680	134.8	125.0	145.3

*NEC* not elsewhere classified

^†^Perioperative events that co-occurred during the initial admission for RP were excluded

^††^Includes anorectal fistula, abscess, prolapse, stenosis, haemorrhage, and ulcer

^#^Includes bladder stenosis and urethral stricture

^‡^Rate per 1000 person-30 days, ^§^Rate per 1000 person-90 days

**Table 4:** Top 10 reasons for rehospitalisation within 30 days of radical prostatectomy (N = 5105)

No	Reasons	N	%
1	Urinary obstruction	163	3.2
2	Haematuria	131	2.6
3	Urinary tract infection	126	2.5
4	Post-procedural genitourinary complications (not elsewhere classified)	103	2.0
5	Incontinence	59	1.2
6	Urinary device complications	56	1.1
7	Haemorrhage/haematoma	52	1.0
8	Functional gastrointestinal changes	47	0.9
9	Anaemia	46	0.9
10	Post-procedural gastrointestinal complications	23	0.5

**Table 5:** Top 10 reasons for rehospitalisation within 90 days of radical prostatectomy (N = 5105)

No	Reasons	N	%
1	Urinary obstruction	273	5.3
2	Urinary tract infection	213	4.2
3	Haematuria	178	3.5
4	Post-procedural genitourinary complications	167	3.3
5	Anaemia	94	1.8
6	Incontinence	93	1.8
7	Urinary device complications	82	1.6
8	Functional gastrointestinal changes	77	1.5
9	Haemorrhage/haematoma	59	1.2
10	Post-procedural gastrointestinal complications	29	0.6

**Table 6:** Top 10 perioperative events during the same admission for radical prostatectomy (N = 5105)

No	Reasons	N	%
1	Incontinence	239	4.7
2	Urinary tract infection	184	3.6
3	Haematuria	150	2.9
4	Post-procedural genitourinary complications	118	2.3
5	Haemorrhage/haematoma	100	2.0
6	Urinary device complications	91	1.8
7	Cystitis	59	1.2
8	Post-procedural circulatory complications	42	0.8
9	Puncture/laceration	37	0.7
10	Medical-surgical complications not elsewhere classified	30	0.6

In the final multivariable adjusted models, adjusted for all covariates, compared with men aged less than 60 years, those in the older categories had higher rates of rehospitalisation within 90 days of RP (aged 75 + vs < 60: incidence rate ratio (IRR) 2.23, 95% CI 1.88–2.64; 70–74 vs < 60: IRR 1.57, 95%CI:1.30–1.88; and 60–64 vs < 60: IRR 1.25, 95% CI 1.05–1.49). High comorbidity burden (Rx-Risk score 3 + vs 0: IRR 2.33, 95% CI1.80–3.01 and Rx-Risk score 2 vs 0: IRR 1.29, 95%CI: 1.08–1.54) was associated with higher rehospitalisation rates. Having a higher PSA at diagnosis (> 20 vs < 10 ng/mL: IRR 1.67, 95% CI 1.34–2.08) or Gleason score 9–10 vs < 7 (IRR 1.39, 95% CI 1.06–1.84) were also associated with higher rehospitalisation rates. In contrast, men who were treated from 2016–2021 (IRR 0.61, 95% CI 0.53–0.71) had lower rehospitalisation rates compared with patients who were treated from 2002–2005 (Table 7).

**Table 7:** Multivariable zero inflated negative binomial regression, rehospitalisation within 90 days of radical prostatectomy

Variables		IRR	95% CI		*p* value
Age at diagnosis	< 60	Ref			
	60–64	1.16	0.96	1.39	0.119
	65–69	**1.25**	**1.05**	**1.49**	**0.012**
	70–74	**1.57**	**1.30**	**1.88**	**< 0.001**
	75 +	**2.23**	**1.88**	**2.64**	**< 0.001**
Socioeconomic status	Lowest (most disadvantage)	Ref			
	Low	1.30	0.83	2.03	0.245
	Average	1.24	0.83	1.84	0.293
	High	1.14	0.73	1.79	0.559
	Highest (least disadvantage)	0.77	0.49	1.20	0.246
Remoteness index	Major city	Ref			
	Inner regional	**0.79**	**0.68**	**0.92**	**0.002**
	Outer regional	0.91	0.79	1.05	0.200
	Remote/very remote	0.83	0.64	1.08	0.162
Place of birth	Australia and New Zealand	Ref			
	UK and Ireland	1.23	0.99	1.52	0.060
	Other	1.13	0.97	1.32	0.112
Year of treatment	2002–2005				
	2006–2010	0.97	0.66	1.43	0.882
	2011–2015	0.72	0.48	1.09	0.117
	2016–2021	**0.61**	**0.53**	**0.71**	**< 0.001**
Rx-Risk comorbidity index	0	Ref			
	1	1.04	0.86	1.26	0.649
	2	**1.29**	**1.08**	**1.54**	**0.005**
	3 +	**2.33**	**1.80**	**3.01**	**< 0.001**
Diagnostic PSA level	< 10	Ref			
	10–20	1.08	0.90	1.31	0.399
	> 20	**1.67**	**1.34**	**2.08**	**< 0.001**
Gleason grade (GG) group	GG1 (score < 7)	Ref			
	GG2 (score 3 + 4)	0.85	0.71	1.01	0.062
	GG3 (score 4 + 3)	1.13	0.86	1.48	0.386
	GG4 (score 8)	1.18	0.87	1.59	0.277
	GG5 (score 9–10)	**1.39**	**1.06**	**1.84**	**0.019**

*PSA* prostate-specific antigen, *IRR* incidence rate ratio, *CI* confidence interval

Significant associations are indicated in bold

There were inconsistent patterns in the association of rehospitalisation with remoteness index; whilst men in inner regional areas had a lower rate of rehospitalisation compared with men in major cities (IRR 0.79, 95%CI:0.68–0.92), those in outer regional and remote areas had no significant difference. Whilst there was a gradient pattern of lower rehospitalisation rates in men in the highest SES quintile, the association was not statistically significant. Likewise, there was no statistically significant association between rehospitalisation and place of birth (Table 7).

## Discussion

In this study, we quantified rehospitalisation rates up to 3 months post-prostatectomy in a large cohort of South Australian men diagnosed with prostate cancer. We also identified factors associated with rehospitalisation. Rehospitalisation within 90 days of RP was associated with older age, multimorbidity, high diagnostic PSA levels and high Gleason score. Conversely, men who underwent RP in more recent years had lower rehospitalisation rates. There was no statistically significant association of rehospitalisation with SES or place of birth. However, patterns of rehospitalisation according to remoteness of residence were inconsistent. Apart from a brief increase in readmission numbers 5 to 8 days post-prostatectomy, admission rates remained relatively consistent over the first 30 days after surgery.

There is significant heterogeneity amongst studies in the reporting standards of post-treatment rehospitalisation due to differences in the type and number of adverse events included. Consequently, it is difficult to directly compare our findings with those of previous studies. Two recent retrospective studies indicated that complications requiring hospital readmission within 30 days occur in approximately 3% of patients [25, 26] and up to 8% according to a recent randomised trial [27]. A study from Sweden shows that the 90-day readmission rate after robot-assisted, retropubic and laparoscopic RP techniques was 9%, 10% and 11%, respectively [28]. In our cohort, we identified a slightly higher frequency of hospital visits following RP than reported in other studies (9% and 13% within 30 and 90 days, respectively). Despite the differences in reporting standards amongst studies, the common postoperative complications that we observed, such as urinary retention and urinary tract infections, are also reported in other studies [29].

Our findings of higher rehospitalisation rates in men diagnosed at an older age, with high comorbidity, more aggressive tumour features, and treated earlier in the study period are consistent with a previous study from Sweden [28]. Interestingly, men who underwent prostatectomy in recent years had a much lower chance of rehospitalisation than those treated prior to 2016. This reduction in rehospitalisation has coincided with the significant increase in the uptake of robotic surgical systems in Australian public hospitals during the last 10 years with RP being the most prolific robotic procedure performed [30]. A recent meta-analysis comparing open to robotic RP demonstrated lower morbidity in the robotic approach with reduced risk of blood loss and transfusion rate, shorter hospital stay, and fewer overall complications, but no significant differences in major complications and postoperative urinary continence recovery [31]. Australian study has also indicated that robotic RP has a significant advantage over open RP in terms of readmission rates [32].

Other factors contributing to reduced rehospitalisation independent of the surgical approach but related to uptake of robotic systems include a more centralised delivery of prostate cancer surgery with smaller regional hospitals referring to larger tertiary centres with the robotic technology resulting in higher volume centres that can achieve better outcomes [33, 34]. Another consideration is since its inception into the public system, robotic-assisted laparoscopic prostatectomy (RALP) has largely been delivered as a consultant-led procedure with proficiency in robotic surgery not part of a training registrars’ curriculum. This has meant that the primary surgeon in the majority of RALP cases may have been more experienced senior consultants which may contrast with other time periods during this study where open RP was considered a key component of a training registrars’ surgical curriculum.

The lower rehospitalisation rates in more recent years could also be attributed to ‘better patient selection’ for RP. This has been reflected in another study using SA-PCCOC cohort [2], which shows that the proportion of men with low-risk prostate cancer undergoing RP has decreased over the years whilst an increased trend of active surveillance uptake. This trend reflects urologists’ growing adoption of active surveillance for men with low-risk and some favourable intermediate-risk localised prostate cancer, with an aim to reduce overtreatment and preserve quality of life, without losing the window of curability [35–37].

Our study also demonstrated that cancer registry data can be used for more than surveillance and cancer control. By linking with other administrative health records, such as hospital admission datasets, population-based cohorts can be created to study treatment outcomes and healthcare use [14, 22, 23, 38–40]. Such studies aid in resource planning and service delivery, evaluating prevention programmes, and improving quality of care [41]. They can also facilitate the development of targeted interventions to reduce hospital readmissions, ultimately improving patient outcomes and healthcare resource utilisation.

Our findings should be interpreted considering the following potential limitations. First, due to its observational nature (i.e. not a controlled study), our study may have bias due to residual confounding. This is particularly important given that surgical outcomes depend on physician and facility factors, as well as patient and tumour factors. For example, a previous study has shown that surgeons and facilities with higher surgical volumes are associated with a lower risk of complications [33]. Second, although men who were treated in public hospitals are likely to return to seek help in the public system where they were treated, there is still a possibility that these men may have sought follow-up care and treatment from private practices. This could potentially result in an underestimation of the rehospitalisation rates reported in our study. Third, we were unable to report numbers of less than six due to a confidentiality agreement with data custodians. This prevented us from reporting some specific adverse events such as deep vein thrombosis (DVT), pulmonary embolism and stoma formation, though these events were very rare in our cohort. Despite the above limitations, this large heterogeneous population-based study, where health interventions are well captured, provides interesting insights that can inform practice. Recent improvement in post-treatment rehospitalisation rates is reassuring for patients and healthcare providers.

## Conclusion

Post-prostatectomy rehospitalisation is a common occurrence, affecting 13% of South Australian patients within 90 days of RP. Patient-related factors such as older age, higher comorbidity burden and advanced disease stage were associated with increased rehospitalisation risk. Notably, a decreasing trend in rehospitalisation rates was observed over time, suggesting potential improvements in surgical techniques and/or postoperative care.

## Data Availability

The linked datasets that support the findings of this study are stored in the Secure Unified Research Environment (SURE) system, where restrictions apply regarding data access, and so are not publicly available. However, data are available from the authors upon reasonable request and with permission from the data custodians.

## Abbreviations

ICD: International Classification of Diseases
IRR: Incidence rate ratio
ISAAC: Integrated South Australian Activity Collection
PSA: Prostate specific antigen
RP: Radical prostatectomy
SACR: South Australian Cancer Registry
SA-PCCOC: South Australian Prostate Cancer Clinical Outcome Collaborative
